# The Depth Estimation and Visualization of Dermatological Lesions: Development and Usability Study

**DOI:** 10.2196/59839

**Published:** 2024-12-18

**Authors:** Pranav Parekh, Richard Oyeleke, Tejas Vishwanath

**Affiliations:** 1 Stevens Institute of Technology Hoboken, NJ United States; 2 K.E.M. Hospital Mumbai India

**Keywords:** machine learning, ML, computer vision, neural networks, explainable AI, XAI, computer graphics, red spot analysis, mixed reality, MR, artificial intelligence, visualization

## Abstract

**Background:**

Thus far, considerable research has been focused on classifying a lesion as benign or malignant. However, there is a requirement for quick depth estimation of a lesion for the accurate clinical staging of the lesion. The lesion could be malignant and quickly grow beneath the skin. While biopsy slides provide clear information on lesion depth, it is an emerging domain to find quick and noninvasive methods to estimate depth, particularly based on 2D images.

**Objective:**

This study proposes a novel methodology for the depth estimation and visualization of skin lesions. Current diagnostic methods are approximate in determining how much a lesion may have proliferated within the skin. Using color gradients and depth maps, this method will give us a definite estimate and visualization procedure for lesions and other skin issues. We aim to generate 3D holograms of the lesion depth such that dermatologists can better diagnose melanoma.

**Methods:**

We started by performing classification using a convolutional neural network (CNN), followed by using explainable artificial intelligence to localize the image features responsible for the CNN output. We used the gradient class activation map approach to perform localization of the lesion from the rest of the image. We applied computer graphics for depth estimation and developing the 3D structure of the lesion. We used the depth from defocus method for depth estimation from single images and Gabor filters for volumetric representation of the depth map. Our novel method, called red spot analysis, measures the degree of infection based on how a conical hologram is constructed. We collaborated with a dermatologist to analyze the 3D hologram output and received feedback on how this method can be introduced to clinical implementation.

**Results:**

The neural model plus the explainable artificial intelligence algorithm achieved an accuracy of 86% in classifying the lesions correctly as benign or malignant. For the entire pipeline, we mapped the benign and malignant cases to their conical representations. We received exceedingly positive feedback while pitching this idea at the King Edward Memorial Institute in India. Dermatologists considered this a potentially useful tool in the depth estimation of lesions. We received a number of ideas for evaluating the technique before it can be introduced to the clinical scene.

**Conclusions:**

When we map the CNN outputs (benign or malignant) to the corresponding hologram, we observe that a malignant lesion has a higher concentration of red spots (infection) in the upper and deeper portions of the skin, and that the malignant cases have deeper conical sections when compared with the benign cases. This proves that the qualitative results map with the initial classification performed by the neural model. The positive feedback provided by the dermatologist suggests that the qualitative conclusion of the method is sufficient.

## Introduction

### Background

Skin cancer is the abnormal growth of skin cells that most often develops due to exposure to UV radiation. Based on the affected cells, the skin lesions caused by the cancer are divided into melanocytic and nonmelanocytic [1]. Nonmelanoma skin cancers are divided into basal cell carcinoma, squamous cell carcinoma, and Merkel cell carcinoma. Basal cell carcinoma is the most common type of skin cancer but is usually treatable. On the other hand, melanocytic skin cancers are divided into melanoma and nevus. Melanoma is a serious skin cancer that can be fatal if not detected early. Melanoma is life-threatening when it grows beyond the skin’s dermis, making depth an essential factor in treating melanoma [2].

Based on how deep the cancer has penetrated the skin, melanoma can be classified into 5 stages. Stage 0 is curable and occurs when the lesion is on top of the skin. Stages 1, 2, and 3 are curable through surgery (or advanced surgery) and medication; however, as the stages increase, so does the difficulty in treating the cancer. Stage 4 is the deadliest of them all, and it occurs when the cancer has spread into lymph nodes and organs. There are low survival rates among patients 1. Therefore, the early detection of melanoma is essential. We want to be able to detect melanoma within the earlier stages [3]. Once melanoma is detected, we want to reconstruct the lesion into a 3D holographic projection to examine the skin lesion’s depth. Analyzing the depth tells us the stage of cancer and the treatment required.

Our first step is to detect melanoma from a given input skin lesion using machine learning (ML) and explainable artificial intelligence (XAI). This study uses ML as an umbrella term for neural models and computer vision. ML is growing rapidly, and its excellent performance has enormous potential in many fields, including health care. However, there is a need to explore the interpretability of ML models. They are commonly used as a black box that throws an output based on a specific input data sample. However, for fields like health care, where context plays a vital role, recent research has been explored to develop XAI. XAI methods help explain the decisions and predictions made by the model. This helps us improve our systems and fix our hyperparameters while implementing the models [4]. In the next section, we shall review some XAI methods and use them to detect skin melanoma.

The second step is reconstructing the detected melanoma lesion as a 3D holographic projection. This uses computer graphics concepts like depth map estimation and surface reconstruction. We also developed a novel method called red spot analysis to quantify the amount of infection with increased depth into the skin. Our final output is a conical structure of the lesion that can be visualized and interacted with as a hologram through a mixed reality (MR) headset. The reason for proposing the use of MR is to blend the real and virtual worlds so that we have a projection of the lesion within a real-world setting [5].

The summary of the major contributions of the paper are as follows: (1) it gives the physician a tool to estimate how much the lesion proliferated into the skin; (2) the hologram is interactive, so certain areas can be zoomed in and studied in detail; and (3) this enables quick and accurate diagnosis of the patient.

Before moving on to the implementation methodology, we review how ML and MR have impacted the treatment of skin lesions and assisted physicians in making decisions.

### ML Models and XAI for Melanoma Detection

The following study uses ML as an umbrella term that includes computer vision and neural networks. The classification of tumors as benign and malignant has been a familiar logistic regression problem [6]. Numerous studies have extended ML algorithms for the classification of skin lesions to detect melanoma [7].

The study by [8] used a computer-aided diagnosis system to classify the 2 classes of skin lesions—benign and malignant. Classification is performed by 4 ML classifiers, which consist of support vector machine, hidden naive Bayes, random forest, and logistic regression. The paper by Hosny et al [9] presents a skin lesions classification system based on transfer learning and neural networks. They use the Alex-Net alongside the softmax activation function for the multiclass classification of 3 types of lesions. They classify the segmented color images into melanoma, nevus, and seborrheic keratosis.

Performing segmentation is often used as a preclassification procedure in several studies. Fernandez Alcon et al [10] performed threshold-based segmentation based on Otsu’s algorithm. The shape, color, and texture features are extracted from the segmentation, which are used in identifying malignant melanoma from Clark nevi. On the other hand, Wighton et al [11] used supervised mechanisms like the maximum a posteriori (MAP) technique for segmentation and G-LoG (Gaussian–Laplacian of Gaussian) for classification. Another standard algorithm for extracting color features before applying logistic regression is k-mean clustering and k-nearest neighbors [12].

Apart from detecting melanoma, it is critical to detect the depth of the cancer. The 3 common characteristics of melanoma moles are as follows: (1) the outer covering of the moles is ragged, asymmetrical, and coarse; (2) almost half of the moles present do not resemble the other half of the moles; and (3) the newly formed moles are of different shape, color, and texture from the previously existing moles.

Based on the features of the moles, we get an idea about the spreading level and severity of the disease. The proliferative index is the fraction of the total active nuclei present at that instance of time [13]. Its relation to the depth of the lesion is yet to be studied. The study by Kumar et al [14] used the sum rule fusion method and artificial neural network to confirm whether the melanoma stage is critical. However, this method does not clarify the distinction between each stage. To find a clear difference between each stage, we need to estimate the depth of the lesion within the skin and lymph nodes.

Although ML models have improved accuracy in melanoma detection, there is a lack of transparency in how these systems obtain their results. XAI systems are used to provide explanations to clinicians, thereby solving the issue of transparency. There are two branches of XAI techniques [15]: (1) intrinsically and inherently understandable algorithms, but there could be a trade-off between performance and interoperability, leading to bad results; and (2) retrospective post hoc algorithms, which are often rejected in the medical field due to the risk of confirmation bias along with the explanations.

Chanda et al [15] developed their own multimodal XAI system that matched the XAI explanations to the clinician’s judgments, aligning it well with the medical task. Deep neural networks like Alex-Net mentioned above have primarily been seen as black-box predictors. Papanastasopoulos et al [16] used XAI techniques like the integrated gradient attribution method and SmoothGrad Noise Reduction algorithm to visualize the model’s contributing features internally.

Recently, convolutional neural networks (CNNs) have achieved excellent results in detecting and diagnosing melanoma [17]. Deep pretrained convolutional models have also been used to extract features from skin lesions for necessary classification [18]. Such models consist of convolutional; pooling; and dense, fully connected layers for the required output. A paper by Zhou et al [19] used the global average pooling layer (GAP) to support the localization of objects in an image. They are used to retain the spatial structure of the feature maps and identify discriminative regions of the image. They performed the GAP operation on the feature maps just before the final softmax activation layer, which helped determine the critical regions of the image. The class activation map (CAM) indicates the discriminative region CNN uses to classify the image into its corresponding class. It does so by projecting back the output layer weights onto the convolutional feature map.

The primary limitation of the CAM method is that architectural constraints bind it: only the architectures with GAP layers before the softmax layer can use CAM visualizations. The modified model must be retrained, which can also slightly trade off the model’s performance. Therefore, this falls under the first category of the XAI techniques mentioned above. A more generalized approach proposed by [20] improved the limitations of CAM. The gradient class activation map (GradCAM) technique considers the target object’s gradients flowing into the final convolutional layer to create a localization mapping that highlights the essential regions of the target image. We use this XAI method to highlight the areas responsible for the classifier’s output.

### 3D Depth Estimation and MR Visualization of the Skin Lesion

Depth estimation is the task of measuring each pixel relative to the camera. Concerning skin lesions, the depth of a pixel relative to the skin surface denotes how critical the situation is for the patient. Depth is extracted from either single (monocular) or multiple (stereo) views of an image. Structure from motion [21], stereo vision [22], and depth from focus and defocus [23] are used to estimate depth considering multiple images. In the following study, we have a singular top view of the skin lesion as our input for depth estimation.

The conventional methods for defocus estimation have relied on multiple images [24]. The defocus is measured using a deblurring process over an image set of the same scene captured using multiple focus settings. On the other hand, with constrained image acquisition techniques like active illumination [25] and coded aperture method [26], we can estimate depth using single images that focus on one view. However, their main drawback is that they require additional illumination and camera modification to obtain the defocus map. In Zhuo and Sim [27], a novel technique is used to estimate the defocus occurrence from a single image. Defocus estimation refers to the depth estimated from a defocus blur at the edges of an image. We obtain a full defocus occurrence map by propagating the defocus blur amount into the inner portions of the image. Using the following concept, we estimate the depth of the lesion from a single image.

Consequently, once we have estimated the depth of the lesion, we want to visualize it as a 3D volumetric structure so that it can be analyzed correctly. The Gabor filter is a linear filter that combines a sine wave with a Gaussian envelope. The combination of orientation with the Gaussian function makes it well-suited for edge detection [28], local feature extraction [29], and texture analysis [30]. We extend the application to 3D reconstruction by using multiple Gabor filters with different orientation and frequency values to capture the range of structural features. Apart from the frequency and orientation, bandwidth is a crucial parameter that is decided based on the characteristics of the image [31].

Once we obtain the 3D structure, we analyze the diagram and calculate lesion volume units within varying depth ranges under the skin. Our end goal from the testing pipeline is to have an interactive holographic projection visualized on an MR headset. Using MR, the physician can analyze the criticality of the melanoma and under which stage the melanoma could be at that time. MR headsets like the Hololens 2 (Microsoft) use the *Mixed Reality Tool Kit* library onto which the holograms are uploaded for visualization [32]. Interoperative navigation has been performed in different surgeries using different extended reality techniques. Recent studies involving navigation through holograms include Porpiglia et al [33] for percutaneous kidney puncture, Kitagawa et al [34] for laparoscopic cholecystectomy, Cai et al [35] for craniomaxillofacial surgery, and Li et al [36] for laparoscopic nephrectomy. Other studies have tested the feasibility of such navigation systems; for example, a study concerning the skin tested the feasibility of MR-based navigation toward the sentinel node in patients with melanoma [37]. In our study, we want to use MR as an analysis tool for estimating the depth of the lesion within the skin.

## Methods

### ML and XAI for Melanoma Detection

As mentioned in the introduction, this is the first step of our study. We detected melanoma from a skin lesion and then used GradCAM as our XAI technique that highlights the essential parameters of the image. We have elaborated on this further in the following section.

#### Dataset and Data Processing

For this study, we used processed skin cancer images from the International Skin Imaging Collaboration (ISIC) archive. The dataset is well balanced, having 1497 malignant images and 1800 benign images. The malignant images primarily included skin lesions that proliferate under the skin and could even have reached the lymph nodes if not treated. In our dataset, nevus and melanoma are malignant were nature. If a particular lesion was labeled as malignant, there was a high chance that it could have been melanoma, making it critical to detect malignancy. We also wanted to give an output expressing the degree of malignancy of the lesion, which told us how close it could be to melanoma.

We first labeled each image as “Benign” and “Malignant” since we wanted a clear idea of what each data point represented during training. Both image categories had the same shape distribution of (224, 224, 3), where the area of the image was 224×224 and 3 represented the red-green-blue value. Therefore, the model was trained on a uniform distribution, not affecting the output label. We then split the dataset into training, validation, and testing sets. After the split, we had 2373 samples in the training set, 264 in the validation set, and 660 in the test set. We used the *LabelEncoder* from the *sklearn* library to convert “Benign” and “Malignant” annotations into categorical labels. Figure S1 in Multimedia Appendix 1 better represented the dataset.

#### Model Architecture

The “SkinCancerDetection_VGG19_Model” architecture comprised 2 levels. First, the base model: we used a pretrained VGG19 model with weights used for the ImageNet dataset [38]. Convolution blocks 1 and 2 had two convolution layers, while blocks 3, 4, and 5 consisted of four. This was designed to capture features hierarchically, where the earlier blocks captured low-level features like edges and textures and the latter blocks captured high-level features. Each block included one pooling layer. The “include_top” parameter was set to false because we wanted to exclude the top layers of the model for this specific task. Second, the functional model: we used a model that used Flatten on the base model’s output and then 5 dense layers with the ReLU activation function. They performed hierarchical feature reduction with each layer. The final dense layer is of one unit, with the sigmoid activation function determining the class of the output label. The functional model is depicted in Figure S2 in Multimedia Appendix 1.

Pretrained models had been trained on large, diverse datasets, imparting valuable knowledge about low-level features. They also helped prevent overfitting since they are generalized on a large dataset like ImageNet. The model loss is calculated as the binary cross entropy, and we used the Adam optimizer for optimization.

#### GradCAM for Localization

**Figure 1:**
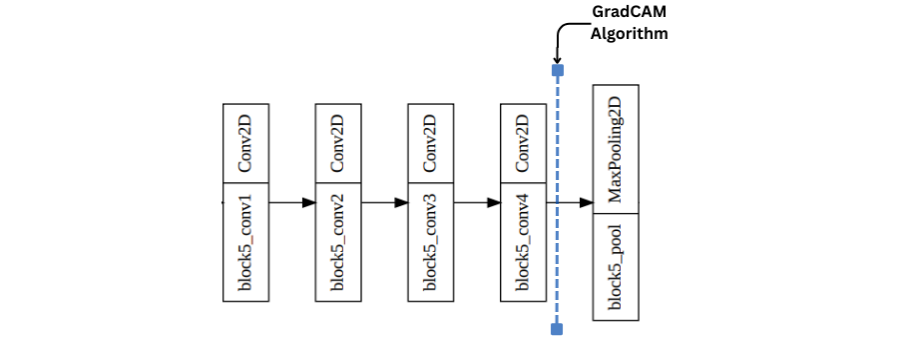
Application of the GradCAM algorithm within the model. Conv2D: Convolutional 2-Dimensional; GradCAM: gradient class activation map.

As discussed in the literature review section, GradCAM is an intrinsically implemented XAI technique used to localize the image’s essential parameters. We used GradCAM instead of standard object detection methods since it provided us with a heatmap of the localized area instead of a bounding box. This heatmap was useful for the depth estimation of the region. GradCAM computed gradients of the target label flowing from the final convolution layer, followed by a weighted sum of the feature maps in the final layer to create a localization mapping depicting the important parameters. In our case, the localized portion was the part of the lesion on the skin. The GradCAM was a well-established method that we implemented in our *GradCamUtility* file. We entered the “block5_conv4” attribute into the *GradCamUtility* class since it was our last convolution layer of the model. The final CAM depicted the feature maps that contributed most to the corresponding output label. A visual representation of the layers within a convolution block alongside where the GradCAM algorithm was applied is depicted in Figure 1. An original image and its corresponding CAM are depicted in Figure S3 of Multimedia Appendix 1.

### 3D Generation of Skin Lesion Depth

As discussed in the introduction, our second step was to generate a 3D hologram that helped physicians analyze the depth of the lesion within the skin. We did this in three steps: (1) estimating depth from defocus occurrence; (2) 3D structure representation of the depth; and (3) red spot analysis.

#### Estimating Depth from Defocus Occurrence

As discussed in the literature review, we used the defocus occurrence method to estimate depth when we have one input image of a particular scene. After GradCAM, we had a blended image with localization of the important parameters as our output. We performed Canny Edge detection on the blended image, which extracts the edges and boundaries of the image, thereby giving us an outline of a localized area. We used the threshold values of 50 and 150 to determine strong, weak, and no edges. The following values gave us the appropriate boundary for the specific image.

We applied the Gaussian blur over the entire image for smoothing since it can help create coherent depth maps and reduce the impact of noise. We applied the defocus occurrence method, with the edges and blurred values being the 2 input attributes of the function. Finally, we used minimum-maximum normalization as our normalization technique on the defocus map. Finally, we had the output defocus map. The algorithm had been performed in the code.

#### 3D Structure Representation of the Depth

We used Gabor filters for the 3D structure representation of the depth [39]. The *getGaborKernel* of the *cv2* (Computer Vision Python) library provided a straightforward way to generate Gabor filters with the required parameters. Tuning hyperparameters was essential for the desirable outcome. Several architectural decisions had to be made while using Gabor filters. The parameters and findings were discussed in Textbox 1.

The 3D output of the Gabor filter was constructed as a scatter plot using the *Plotly3D* library in Python. We got a 3D heatmap on the “RdBu” color scale, where the red spots denoted the deeper-lying area of the lesion. We then introduced the red spot analysis to estimate the depth of the corresponding lesion and constructed a 3D conical structure for the same.

**Parameters and findings of 3D structure representation of the depth.**

- Number of Gabor filters: We used 4 at different orientations to construct a 3D structure from the 2D defocus depth map.
- Orientation: We used Gabor filters from [0, pi/4, pi/2, 3×pi/4] to analyze textures from different orientations. For 0 degrees, the textures are analyzed horizontally, while for 90 degrees, the textures are analyzed vertically.
- Frequency: Frequency was an important parameter since it determines if we want to capture finer details. We set the frequency values for the 0- and 90-degree filters as 0.1 since we want the finer details horizontally and vertically. We have set the frequency for the angled filters at 0.4 since we want the filters to capture the coarse details.
- Sigma value: The sigma value represents the sigma of the Gaussian distribution. It represented the blur along a particular direction. We selected a low value of 0.01 along both axes for a sharper, pixelated output.
- Gamma value: We selected a low gamma value of 0.5, making the output anti-isotropic for finer texture analysis.
- Size: We selected a Gabor filter of dimensions (5,5). It was a trade-off size recommended for capturing the coarse and fine details of the image.

#### Red Spot Analysis

The output from the Gabor filter had been constructed using the “RdBu” color scale. The “RdBu” scenario contrasted 2 extremes where the red values indicated areas with higher distances from the viewer, and the blue regions were closer to the imaging device. For a given depth *d*, the spots above it were blue, while those below it were the red spots. These red spots were not literal spots present on the skin caused by secondary infections but were considered spots in the depth map beneath the skin surface. The red spots quantified the amount of infection since a malignant lesion could have red spots over greater depths as compared with benign cases. We checked the red spots for consecutive depths and how much they decreased as we went deeper into the skin. This showed us how the number of red spots decreased with each depth range, and we could note where there were 0 red spots, which signified the end of the lesion. A sample study of the red spot analysis was done for 4 cases depicted in Table 1. We have 2 cases that were benign with lower probabilities of malignancy and 2 cases that were malignant. It showed a change in the number of red spots with depth for all 4 cases. “Below 100” represented the depth on the color map, and the corresponding values were the red spots that proliferated deeper than 100 units. In contrast, 0 represented the skin’s surface.

The observations from the depicted red spot analysis are in Textbox 2.

The final step in the pipeline was to represent the red spot values for each depth range as conical slices. These conical slices, when connected, represented a 3D conical structure of the lesion beneath the skin. We developed a code that took the red spot values for a test case as our input and gave the 3D conical structure as output. The 3D conical structure intended to depict the depth of the lesion and how much it had proliferated within the skin. A physician could view the structure as an interactive hologram on the MR headset and determine the depth and stage of the skin cancer.

**Table 1:** The red spot analysis.

Depth threshold	10% Benign	98% Malignant	2% Benign	99% Malignant
Below 0	176770	180211	178262	176263
Below 100	1809	35059	2605	21288
Below 200	470	12925	1222	11870
Below 300	235	4208	588	6573
Below 400	91	1132	283	3414
Below 500	27	211	126	1745
Below 600	2	19	6	728
Below 700	0	4	0	254
Below 800	0	0	0	132
Below 900	0	0	0	51
Below 1000	0	0	0	0

**Observations from the red spot analysis.**

- We observed that the red spots number decreased as we went deeper within the skin. This was because the lesion volume was more significant in the upper layers of the skin.
- Once we got 0 red spots, we could say that the lesion had not surpassed that particular depth threshold. For example, the first test case did not have spots above 700, signifying that the lesion depth was less than 700 units of the heatmap.
- For the malignant cases (higher probability), we noticed a significantly higher volume of spots beneath the skin depth of 100 units and onwards.
- We also saw that malignant tumors were deeper and could extend to “Below 900” depth units as seen in the fourth test case.

### Ethical Considerations

Although the following research involves human records, that is, images of lesions on their bodies, the dataset used is widely known and used by a lot of researchers. We use the dataset formed by the ISIC that collects and sorts these images while maintaining the privacy of the human [40]. There is complete approval to use this dataset after citing the source.

## Results

### Overview

In this section, we display the evaluation metrics of the model on the testing data and the results from the testing pipeline output. We consolidate and summarize all the required outputs from the experiment.

### Model Evaluation

**Figure 2:**
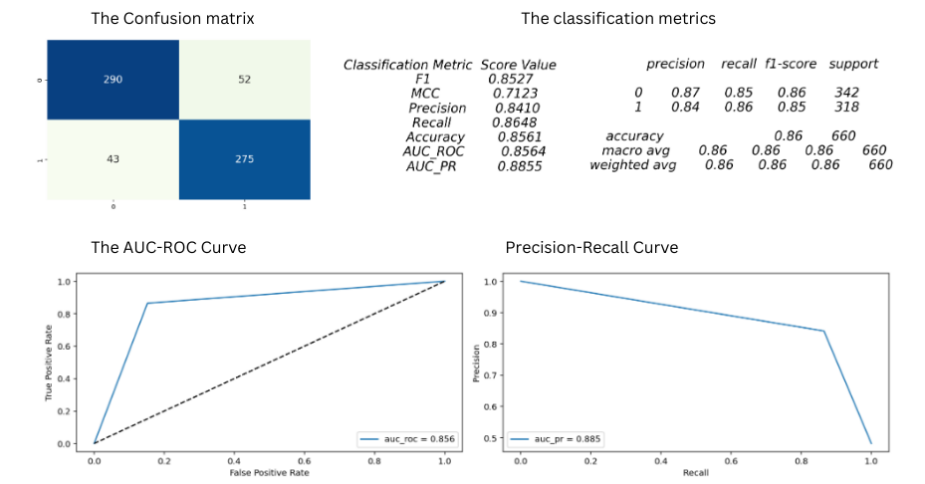
The figure represents the evaluation metrics of the model. The confusion matrix gives us the TP, FP, FN, and TN values, allowing us to calculate the other metrics. The AUC-ROC curve represents the TP-FP trade-off, and the AUC-PR curve represents the precision-recall trade-off. AUC-PR: area under the precision-recall curve; AUC-ROC: area under the receiver operating characteristics curve; FN: false negative; FP: false positive; MCC: Matthews correlation coefficient; TN: true negative; TP: true positive.

As a part of the evaluation, we display the confusion matrix for each label, which gives us the true positive, false positive, true negative, and false negative values. We calculated the precision, recall, and *F*_1_-score based on the matrix’s values. We calculated the Matthews correlation coefficient, which is more reliable since it gets a high score only if the prediction obtains satisfying results in all four categories of the confusion matrix. The Matthews correlation coefficient is a more informative score in evaluating binary classifications than the accuracy or *F*_1_-score. We also studied the area under the receiver operating characteristics curve, which represents the true positive–false positive trade-off, and the area under the precision-recall curve, which represents the precision-recall trade-off. The evaluation metrics for the model are depicted in Figure 2.

The conclusions about the VGG19-GradCAM model based on the evaluation scores are mentioned in Textbox 3.

It is important to state that we have yet to record the trade-off in the metrics due to the addition of the GradCAM calculations after the final convolution layer. There may have been a mild decrease in the recorded metrics due to the presence of these manipulations. Our goal for this experiment is to estimate depth through the 3D reconstructions of the lesion.

**Conclusions about the VGG19-GradCAM model based on the evaluation scores.**

- We had a Matthews correlation coefficient score of 0.71. Since it ranges from 0 to 1 where 0 represents random guessing and 1 represents perfect prediction, a score of 0.71 is good since it indicates a strong positive correlation between the model predictions and class labels.
- The accuracy depends on the correctly labeled data (true data), and a score of 0.86 is satisfactory.
- The precision, recall, and *F*_1_-score depend on the false and true data points, and all three are satisfactory values. We had a precision value of 0.84 and a recall of 0.86. The *F*_1_-score represents the harmonic mean (balance) between the 2 values, taking the value of 0.85.
- The receiver operating characteristics and precision-recall scores were calculated from their respective curves, taking the values of 0.86 and 0.89. Since our dataset is mildly imbalanced toward the negative class (0 or benign), they are essential scores.

### Results from the Pipeline

**Figure 3:**
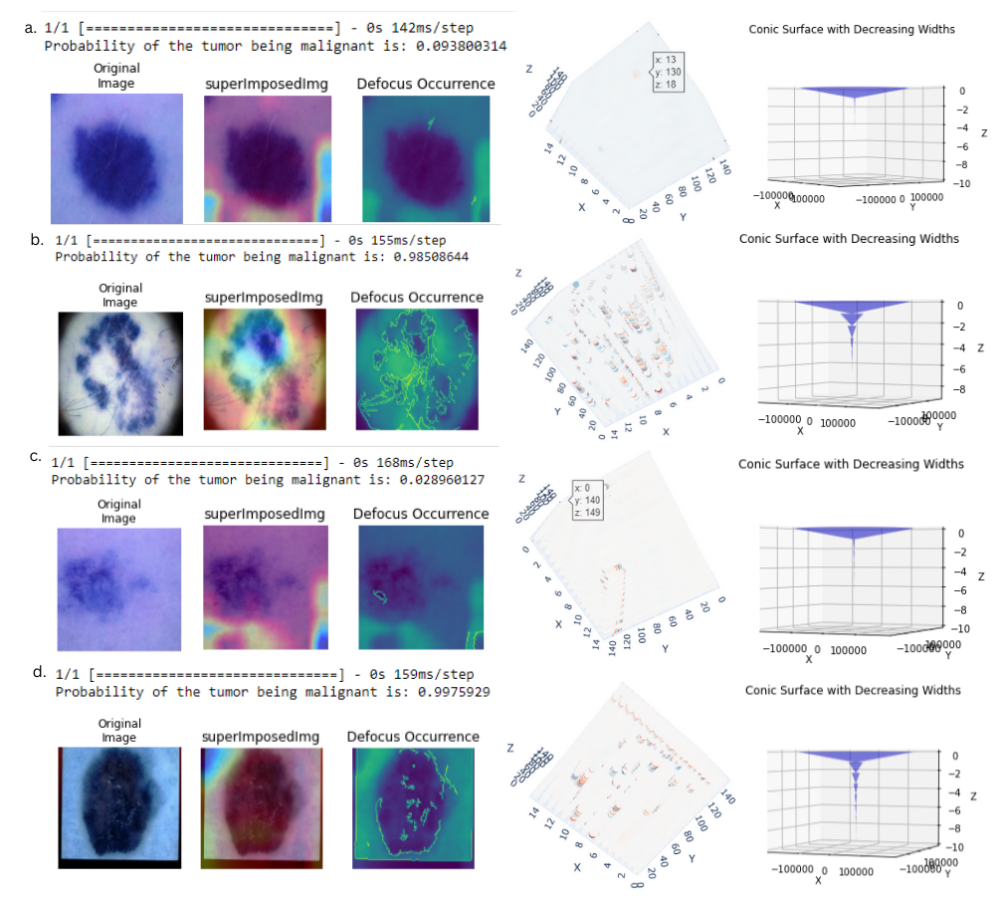
The figure represents the output of the testing pipeline. We have the original image, the image after localization, the depth map, the 3D representation of the map, and the conical representation of the hologram (left-to-right).

We display the end-to-end output of every step mentioned in the study in Figure 3. The 4 cases correspond to those in Table 1 since we have performed the red spot analysis for them. We trained the model over the single test data point and got the probability of malignancy as the output. We applied GradCAM on the original image, which gave us the “superImposedImg” as the output and performs depth estimation, giving us the defocus occurrence map as shown on the “viridis” color map. The 3D output of the depth was obtained after using the Gabor filter. After the red spot analysis, we got the final 3D conical representation of the lesion “Conic Surface with Decreasing Widths.” The decreasing widths represent the number of red spots greater than a certain depth unit.

The conclusions based on the outputs of the testing pipeline in Figure 3 are mentioned in Textbox 4.

The 3D conic surface and depth map were visualized on an MR headset. The hologram was interactive, and we managed to see a representation of the lesion. We see the depth with its corresponding units beneath the skin, and we can also compare multiple such holograms. The generated hologram looks considerably similar in its 3D format to that of the image in the testing pipeline. We consulted a physician for the same since he could provide better intuition concerning the results of the experiment. Upon collaboration, we present the dermatologist’s feedback in the following section.

**Conclusions based on the outputs of the testing pipeline.**

- Nonmalignant cases: A lighter-colored lesion is not heavily distinguished from the rest of the skin. We did not have a significant defocus occurrence boundary as seen in the cases of Figure 3A and C. Due to the lack of a boundary, our 3D representation was mildly covered by red or blue spots. Most spots were neutral, and our conic surface was heavily represented between the 0 and –2 z values.
- Malignant cases: They were better distinguished by the gradient class activation map (GradCAM) algorithm, and their defocus map takes a specific boundary-like structure, as seen in cases Figure 3B and D. The 3D representation of the depth showed a significantly greater number of colored spots. The conic structure had a significant concentration after the –2 z value, extending to a greater depth than the other 2 cases.
- The Figure 3D case had an additional black outline, so we discarded the line of red spots between the range of 135 < y < 145. Such an anomalous line can give us faulty output and more red spots than there are.

### Dermatologist Feedback

The dermatologist, TV, and his colleagues at the institute stress the importance of ML and extended reality for the future of diagnostic imaging. He states that the development of digital health care is alongside applications in the two fields. Although most work is in the research stage, he expects an increase in usage within the next 2 to 3 years. Upon visualizing the depth hologram and discussing research, the dermatologist could comprehend the importance of such a method. He even suggested that he is open to collaborating on such a method, even at their hospital after thorough evaluation and effective examination. He sees a lot of scope clinically, especially since the methodology will give us a tangible method to diagnose lesion depth. He suggests that patients can also use a working application to assess skin lesions unassisted in everyday scenarios.

To evaluate the methodology, he suggests the use of this technique on images from alternate datasets like the Human Against Machine with 10000 Dermoscopic Images (HAM10000). Such datasets include complex classification requirements due to the inclusion of basal cell carcinoma, squamous cell carcinoma, and Merkel cell carcinoma. The methodology must function efficiently for such cases as well. It is important to compare lesion depth scenarios with biopsy reports since they are considered the gold standard in imaging diagnostics. An analogy between the two methods will further solidify the stance of this implementation and what it is trying to achieve. After evaluation, this method can also be unified with dermoscopy to get a paired output comprising processed clinical images and corresponding depth holograms. We plan to collaborate on the evaluation of this method since he believes it could revolutionize the diagnosis of skin lesions.

He recommends generalizing this method to other health care situations where depth estimation is crucial. Filling is an important procedure in aesthetics to hydrate the skin surface. It is crucial to avoid blood vessels since errors in judgment can lead to side effects like strokes, blindness, etc. The use of segmentation and depth maps can be used to locate blood vessels using segmentation and 3D visualization. Angiosarcoma, Kaposi sarcoma, and dermatofibrosarcoma protuberans are rare and aggressive skin cancers that present as a bruise-like purplish lesion. This can be a useful tool as current diagnostic procedures have faltered in lesion diagnosis. This method can also be used for depth estimation in cases like warts, seborrheic keratoses, hypertrophic lichen planus, psoriasis, systemic sclerosis, and morphea. We plan to collaborate on the methodology to improve clinical outcomes and enhance patient diagnosis in depth-related scenarios.

## Discussion

### Conclusions

In this study, we have provided a qualitative methodology for the depth estimation of skin lesions. We have managed to output a hologram that can be visualized by a physician, for diagnosing the patient accurately. We have elaborated the entire pipeline with an output after each step. We have used the initial classification outputs as qualitative evaluation for the generated holograms. We have observed that lesions classified as malignant have greater depth and concentration than nonmalignant cases.

Proceeding this, work needs to be done for the quantitative evaluation of the generated hologram, as this can prove to be a stepping-stone in skin cancer research. We have developed a pipeline that starts with the classification and localization of the lesion. We have used computer graphics to derive the depth map and get a volumetric representation of the lesion. We have developed the red spot analysis to derive the extent of infection within each layer beneath the skin. Finally, we can map the malignant cases to have a greater depth and concentration of infection for each layer of depth beneath the skin. Despite being effective in its estimation, there are a few limitations to overcome in future research.

Very few existing literatures exist for the depth estimation of the lesions. One such paper that we were inspired by discusses the criticality of a particular lesion on the basis of its width, color, and texture [13]. They were trying to determine the depth based on the criticality. They managed to perform melanoma staging using deep learning. We wanted to build up the same by providing a more direct approach to determining lesion depth. We hope that further work can be done to build upon this particular study as well.

### Limitations and Future Work

The study comprises computer vision, graphics, and an MR headset, resulting in a few limitations along the pipeline. Concerning the vision model, we have yet to quantify how much the GradCAM algorithm has affected the model performance. If the difference is significant, we must consider using GradCAM++ or other alternate segmentation methods. Although computer models have achieved a higher accuracy during melanoma detection, it would be the next step to match such accuracy while using XAI methods alongside the model. Apart from that, it is common knowledge that datasets like ISIC are skewed toward lighter skin tones, potentially impacting the application of this methodology on darker skin tones. We need to expand the dataset to contain a more diverse range of skin lesion images, particularly images of color present on the Fitzpatrick Skin Scale. The model can then be trained in an unbiased manner and can be used accurately on all individuals.

The estimation of depth using the defocus method heavily relies on the color and width of the lesion as seen on the skin surface. Despite being malignant, we may not get an appropriate depth map output if our lesion color closely resembles the skin tone. This method also needs to extend to cases where the image is zoomed out, and we can see the entire body part on which the lesion is present. For example, one test case had the presence of a thumb on which the lesion was present, resulting in a slightly inaccurate depth output. We get additional red spots due to the presence of the thumb, making the lesion deeper than it is. Another point of consideration is the skin tone of the person having the lesion. We would need to consider if a darker skin tone projects an increased number of red spots for a similar type of lesion [41].

As computer science researchers, it would not be our place to state the importance of this study in a practical setting. To extend this research to practical applications, it would be crucial to know a physician’s opinion of the hologram when visualized by them through the MR headset. Apart from that, it is also essential to evaluate the generated holograms on the MR headset. For its evaluation, further research must be performed to analyze the validity and fidelity of the rendered hologram. Upon evaluation of the method, this study can have significant implications for melanoma treatment. While previous research [42] provides a web-based tool for early skin cancer risk assessment, this study furthers the field as an accurate model for melanoma staging. Thus, this study can have significant implications for melanoma diagnosis and treatment.

## Appendix

Multimedia Appendix 1 Additional figures.

## Abbreviations

CAM: class activation map
CNN: convolutional neural network
GAP: global average pooling
G-LoG: Gaussian–Laplacian of Gaussian
GradCAM: gradient class activation map
HAM10000: Human Against Machine with 10000 Dermoscopic Images
ISIC: International Skin Imaging Collaboration
MAP: maximum a posteriori
ML: machine learning
MR: mixed reality
XAI: explainable artificial intelligence
