# Supplementary material for: The Depth Estimation and Visualization of Dermatological Lesions: Development and Usability Study
Source: JMIR Dermatol. 2024 Dec 18;7:e59839. doi: 10.2196/59839 (PMC11694055; doi:10.2196/59839)
Supplement: Multimedia Appendix 1 [file derma_v7i1e59839_app1.docx]

Figure S1 depicts the summary of the dataset after preprocessing. Present in the Methods 🡪 ML + XAI melanoma detection 🡪 Dataset and Data Preprocessing section in paragraph 2.


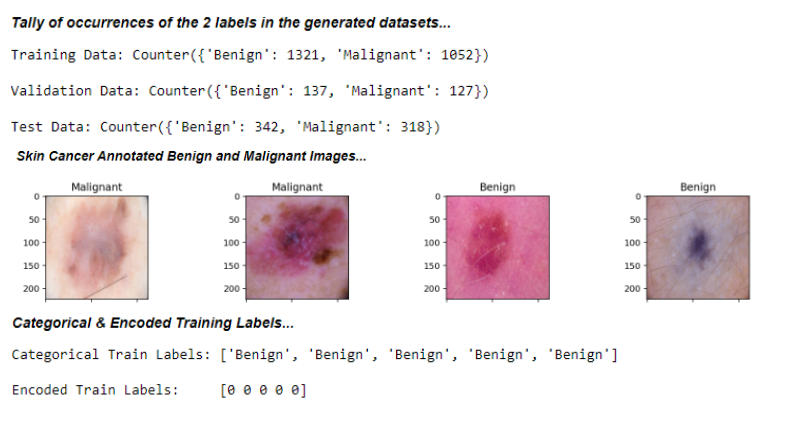


**Figure S1.** The figure gives us the Dataset summary. It gives us the number of samples in the training, validation, and test dataset. We represent the image with annotations and categorical labels.

Figure S2 depicts the functional model trained over the pretrained VGG19. It is responsible for making the final lesion classification: benign or malignant. It is present in Methods 🡪 ML + XAI melanoma detection 🡪Model architecture in point number 2.


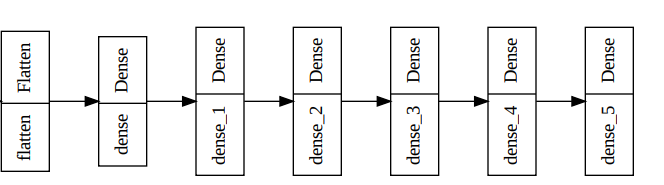


**Figure S2.** The following model is added over the pretrained VGG19 model to extract features efficiently.

Figure S3 depicts the original image with its corresponding GradCAM activation map. Present in Methods 🡪 ML + XAI melanoma detection 🡪 GradCAM for localization under paragraph 1.


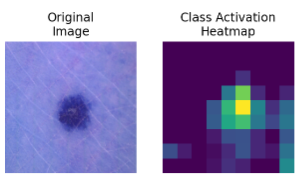


**Figure S3.** An Image and its Activation Map.
